# Estimating Cost of Road Traffic Injuries in Iran Using Willingness to Pay (WTP) Method

**DOI:** 10.1371/journal.pone.0112721

**Published:** 2014-12-01

**Authors:** Elaheh Ainy, Hamid Soori, Mojtaba Ganjali, Henry Le, Taban Baghfalaki

**Affiliations:** 1 Safety Promotion and Injury Prevention research center of Shahid Beheshti, University of Medical Sciences, Tehran, Iran; 2 Faculty of Mathematical Science, Department of Statistics, Shahid Beheshti University, Tehran, Iran; 3 Transport Modeling at AECOM, Melbourne, Australia; Örebro University, Sweden

## Abstract

We aimed to use the willingness to pay (WTP) method to calculate the cost of traffic injuries in Iran in 2013. We conducted a cross-sectional questionnaire-based study of 846 randomly selected road users. WTP data was collected for four scenarios for vehicle occupants, pedestrians, vehicle drivers, and motorcyclists. Final analysis was carried out using Weibull and maximum likelihood method. Mean WTP was 2,612,050 Iranian rials (IRR). Statistical value of life was estimated according to 20,408 fatalities 402,314,106,073,648 IRR (US$13,410,470,202 based on purchasing power parity at (February 27^th^, 2014). Injury cost was US$25,637,870,872 (based on 318,802 injured people in 2013, multiple daily traffic volume of 311, and multiple daily payment of 31,030 IRR for 250 working days). The total estimated cost of injury and death cases was 39,048,341,074$. Gross national income of Iran was, US$604,300,000,000 in 2013 and the costs of traffic injuries constituted 6·46% of gross national income. WTP was significantly associated with age, gender, monthly income, daily payment, more payment for time reduction, trip mileage, drivers and occupants from road users. The costs of traffic injuries in Iran in 2013 accounted for 6.64% of gross national income, much higher than the global average. Policymaking and resource allocation to reduce traffic-related death and injury rates have the potential to deliver a huge economic benefit.

## Introduction

More than 91% of global traffic fatalities occur in low and middle-income countries, which possess only 48% of the world's registered vehicles [1]. Traffic accidents causing injuries have an annual occurrence rate of 26·5 cases per 100,000 people in Iran, and are the country's second-largest cause of death and largest cause of years of life lost (YLL). The proportion of YLL due to traffic injuries in Iran is higher than in most other parts of the eastern Mediterranean region and elsewhere in the world, and is one of the country's most serious problems [2]. Traffic accidents killed three people per 10,000 vehicles worldwide in 2002, while in Iran the rate was 7.3 people per 10,000 vehicles, and this has increased every year [3]. As well as causing pain and suffering, road injuries can push victims' families into poverty due to the costs of medical care, rehabilitation, burial costs, and loss of income. Moreover, traffic injuries put substantial pressure on Iran's healthcare system.

Several studies have calculated the cost of traffic injuries in Iran, each using different methods, but most often the human capital or legal compensation approaches. The cost of traffic injuries using the human capital method was estimated at 180,000 billion Iranian rails (IRR, equal to US$ 6,000,000,000) in 2012 [4]. The human capital approach underestimates the actual cost of injuries due to under-reporting by police, forensics medicine organizations, and insurance companies, and omission of cost components such as lost output, decreased quality of life, and the costs of caring for injured victims and their elderly relatives and children [5].

In contrast, the willingness to pay (WTP) method produces an accurate estimate of cost and is an appropriate way to estimate of total cost makes it easier for politicians to reduce the number of traffic accidents and address the associated problems [6]–[9]. The deaths of approximately 250,000 Iranians during the past 10 years, and the injuries and disabilities caused in millions more people during this time [10] make traffic injuries one of Iran's most important health priorities.

We used the WTP method to estimate the annual cost resulting from traffic injuries in Iran. This method is commonly used in high-income countries but has been less frequently applied in low and middle-income countries due to unavailability of the required information. We aimed to produce the first WTP-based estimate of the cost of traffic injuries in a low or middle-income country, and propose an appropriate model of WTP for Iran.

## Methods

In a cross sectional study on costs resulting from traffic injuries, global report of road safety was reported for Iran 2013 [11], in which the portions of pedestrians, two-wheeled motorcyclists, occupants of four-wheeled vehicles, and drivers of four-wheeled vehicles were 28%, 23%, 26%, and 23%, respectively. Totally 846 (SD = 5, d = 0.05, z = 1.96 and power = 80%) people per road user were randomly selected based on the mentioned percentages from all Tehrani road users and investigated. “*Two main methods have been used to evaluate the benefit of preventing from road traffic injuries: human capital or lost output method and willingness-to-pay method.*

*Human capital or gross output method: The main component in this ex post approach is the discounted present value of the victim's future output which is forgone due to death. This approach has clear disadvantages, since it focuses only on the economic effects of the life loss and does not account for the value and enjoyment of the forgone life, which grossly underestimates the true value of preventing from road crashes and will produce significantly lower values than an ex ante estimate based on willingness to pay. The estimated cost by human capital method is much less than the values derived from willingness-to-pay method*.

*Willingness-to-pay (WTP) approach: This ex ante approach involves some assessment of risk and willingness of individuals to commit resources in exchange for reducing this risk to an acceptable level. However, despite the difficulties associated with the accurate estimation of a person's willingness to pay, it is generally accepted as the most valid method for assessing the value of prevention from road risk. Willingness-to-pay is the preferred methodology, since human capital approach is not conceptually sound. Cost of road traffic injuries in Iran was estimated by human capital method in 2011. Willingness to pay method was first used in a study for precise estimation of road traffic injury cost by the researcher in Iran in 2013* [12], [13]”

The research questionnaire was prepared considering perceived risks and effective variables on willingness to pay. The study questionnaires included three parts: The contingent value (CV) approach involved direct questions; subjects were straight asked that how much they were willing to pay for fatality risk reduction. The stated preference (SP) involved hypothetical scenarios for all road users (public transport drivers, motorcyclist, pedestrians and occupants separately). The Revealed preference (RP) method elicits value from real evidence such as importance to vehicle safety based on his willingness to pay to more safety to his family or added more safety device to own vehicle. In addition WTP approach was used to estimate the statistical value of life and cost of injuries. Inclusion criterion was having at least high school education and being in the age range of 18–65 years old. This project has been approved by ethic committee of Shahid Beheshti University of Medical Sciences on April 21^th^, 2013. After a brief explanation about the study by face to face interview, consent letter was obtained from the subjects. First, demographic questions and then questions about willingness to pay were collected in different scenarios for each type of road users. For precise implementation of the research, a briefing session was held for the interviewers to get them familiar with how to fill out the questionnaire. The collected data were analyzed after their strict control. Final analysis of willingness to pay was carried out using a Weibull model and R software (ver. 2013-03-01) by Institute for Statistics and Mathematics, Vienna.

### Descriptive illustration

**Figure 1:**
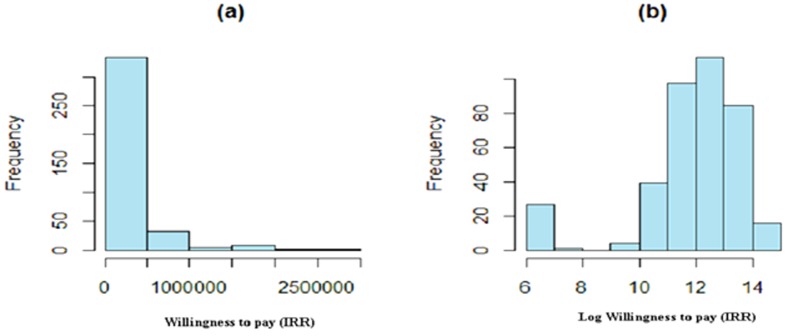
Histogram of willingness to pay (IRR) for drivers (a), Histogram of logarithm of willingness to pay (IRR) for drivers (b).

Fig. 1 shows the WTP (panel a) and logarithm of WTP (panel b) for car drivers as a one sample of procedure. This figure illustrates the severe skewness of the observations. Panel (b) of this figure shows that some people have zero willingness to pay (as the logarithm of 0 is infinity, these zeros are changed to be 500(5,000 IRR) and then the logarithm of 500(5,000 IRR) is calculated). The same patterns are found for other road users (Fig. 1).

**Figure 2:**
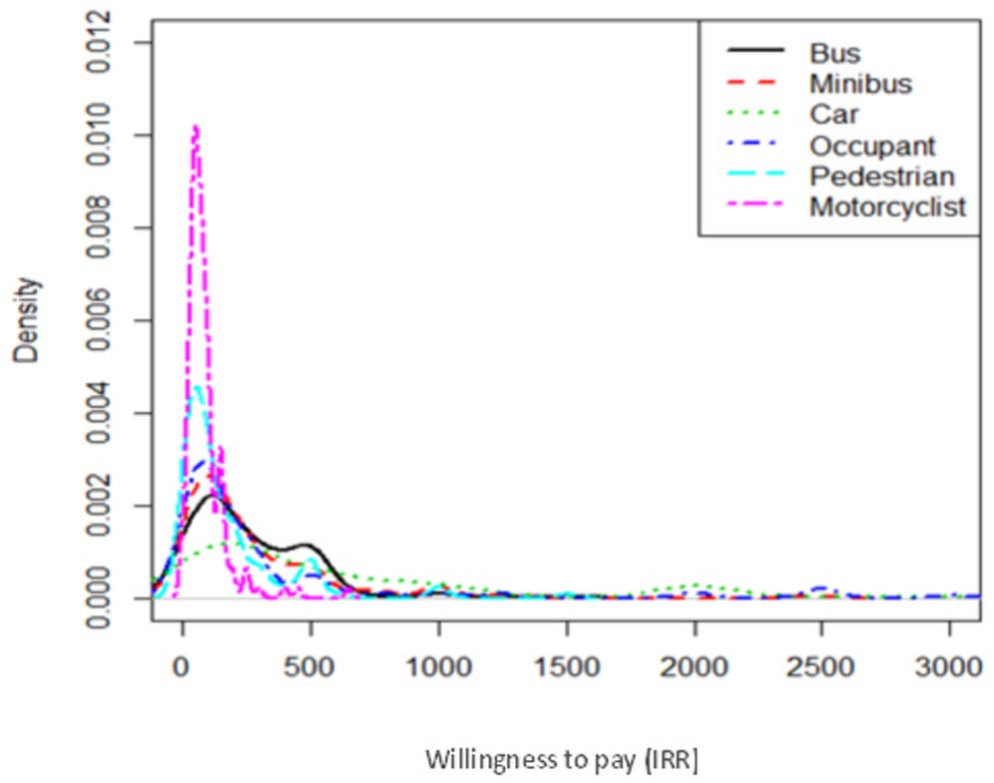
Willingness to pay (IRR) among all road users.

Fig 2 shows the Kernel density diagram for all road users. Bus drivers and motorcyclists have maximum and minimum WTP, respectively (fig. 2).

**Figure 3:**
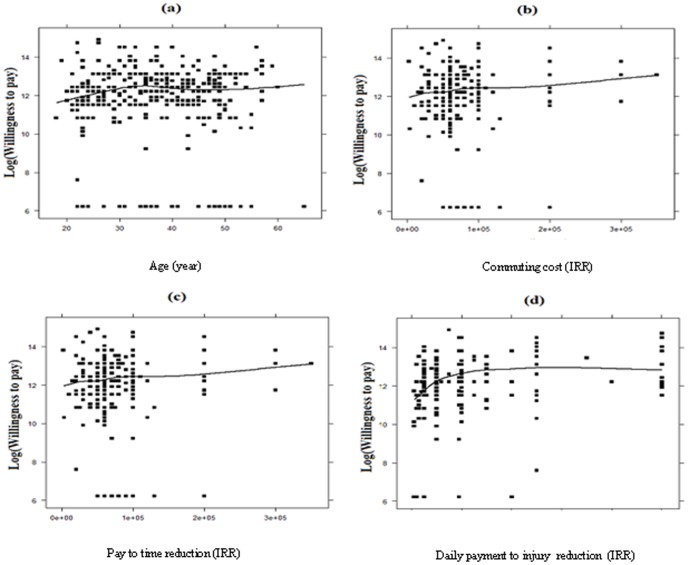
Effects of several variables on WTP (IRR). (a) shows that mean WTP increased up to 35 years old; it gradually reduced from ages 35 to 55 and increased again after the age of 55. (b) shows that people with higher commuting cost had higher willingness to pay. (c) shows that those who paid more to time reduction had higher willingness to pay. (d) shows that people who gave more to charity had higher willingness to pay.

Fig. 3 shows the effect of several variables on WTP (IRR). (Fig. 3)

### Statistical Models for drivers

To model WTP for drivers, a Weibull model was used as follows:
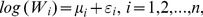
(1)in which
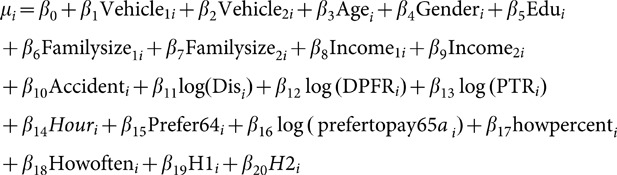



Where Wi is the annual willingness to pay for the i^th^ person and 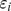 is the model error with extreme exponential distribution (the consequently lead us to have a Weibull distribution for W_i_).

We defined dummy variables by assuming that a categorical variable has three levels (type of car). Thus, two indicator variables were constructed as follows:
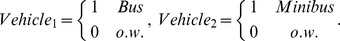



If both indicator variables Vehicle_1_ and Vehicle_2_ have a value of zero, the fact that individual has the third type of vehicle (i.e., private car).

In the above model, age is a continuous variable and gender is specified as the following dummy variable.
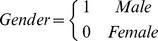



Edu is the indicator variable for education, so that 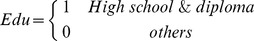



Familysize_1_ and Familysize_2_ are dummy variables of the number of family members and were defined as:
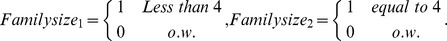



Income_1_ and Income_2_ are dummy variables of income level, defined as follows:
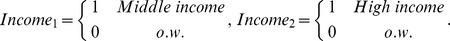



Accident (accident history) is an indicator variable, so that
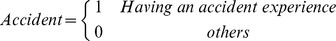



Health status is defined as: 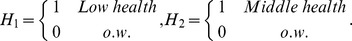



Also, in this model Dis means the distance traveled by an individual (km), DPFR shows daily payment for reducing death risk, and PTR indicates payment for travel time reduction. The other covariates are the number of hours worked per day as a continuous variable and more pay to less traffic, more pay to free flowing traffic, percent of more pay to less traffic, and how often the seat belt was fastened.

### Form of the likelihood

The probability density function of the Weibull distribution with parameters 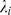 and 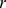 is given by:
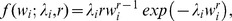



Where 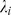 is the scale parameter for the i^th^ individual and r is the shape parameter.

In the Weibull regression model, 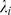 is parameterized again in terms of predictor variables and regression parameters (or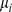) in the following way:
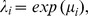



The mean of this model (mean WTP) is defined as follows:
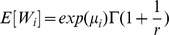



Therefore, the observed likelihood function is as follows:
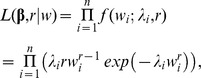



Where 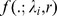 denotes the density function of the Weibull model with shape parameter 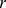 and scale parameter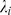.

### Presence of participants with zero willingness to pay: approaches and likelihood

One problem in the analysis of the abovementioned data is the existence of 27 zeroes in the values of the WTP variable. Two solutions exist for this problem. The first method is to replace zero values with the midpoint of 0 and 1000 (10,000 IRR), i.e. 500(5,000 IRR). If an indicator variable is defined as follows:
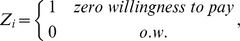



The likelihood function is as follows (assuming *n* as the total number of drivers):
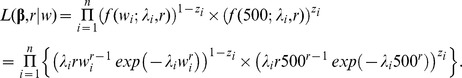



Where 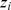 is the observed value of 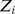.

The second approach for dealing with the zero points is to assume that these people's WTP was less than the minimum value in the sample 1000(10,000 IRR). In this case, the likelihood function is as follows (assuming *n* as the total number of drivers):
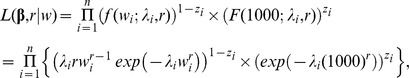



Where f 1000(10,000IRR) is the cumulating distribution function of Weibull evaluated of 1000(10,000IRR).

A general model of WTP for all road users

The risk reduction variable was collinear with the vehicle variable; to eliminate multicollinearity, one of these variables had to be removed from the model. Thus, the two following models were considered.

Model 1: In this model, risk reduction (RR) variable was entered into the model:
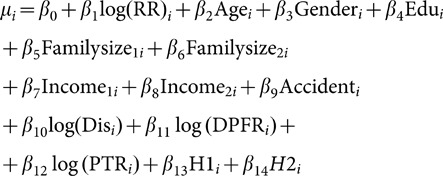



Model 2: In this model risk reduction is omitted but the vehicle variable is included.



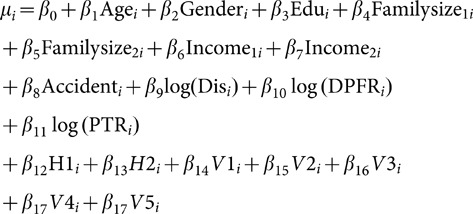
In model 2, indicator variables V1, V2,…, V5 were defined as follows:
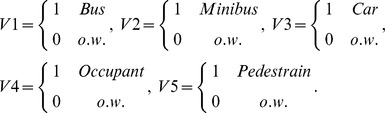



## Results

Of 1000 potential participants who provided informed consent, 846 returned complete questionnaires out, giving a response rate of 84·6%.

Given that one of the key questions of this research was risk perception, it was observed that 64 out of 846 people wrongly responded to risk perception. Therefore, they were removed and data of 782 samples were analyzed. The mean age of the participants was 33·4±9·9 years. 89·3% of subjects were men. Mean family size was 4·25 people. Over half (54·5%) of the studied population was married and 57·3% were main breadwinners. Nearly half (45·8%) of respondents were self-employed, 64·2% owned their homes, and 57·3% owned cars. Just under half of the respondents had a school diploma (375; 48·0%). Maximum percentage of monthly income was between 5 and 10 million IRR (357; 45·7%) and minimum percentage was between twenty million IRR and higher (9; 1·2%). Most of our participants' trips were for business purposes (80·9%). Mean reported cost of monthly commuting was 776·32 IRR. The cost of the statistical value of a life in 2013 was estimated to be 19,713,584,609 IRR, based on mean WTP among road users of 2,612,050 IRR and a reduction in the risk of death from 26·5 per 100,000 people to 13·25 per 100,000 people. The estimated cost of all road fatalities in 2013, based on 20,408 deaths (26·5 death cases per 100,000 people), was US$13,410,470,202. The total estimated cost of non-fatal injuries acquired in road accidents was US$25,637,870,872. The total estimated cost of traffic injuries and deaths in Iran in 2013 was US$390,048,341,074.

Table 1 shows the components of contingent value, stated preference, and revealed preference among the road users. Willingness to pay to have a safer vehicle was higher than WTP for risk reduction (Table 1).

**Table 1:** Components of contingent value, stated preference, and revealed preference among the road users.

Variables	Mean	SD	Min	Max	Median	Mode
**Daily payment (charity)**	31,030	42,040	4,000	400,000	20,000	20,000
**Risk reduction 50%**	2,194/140	3,915,820	0	50,000,000	1,000,000	1,000,000
**Willingness to pay to risk reduction**	2,612,050	3,690,560	0	30,000,000	1,250,000	1,000,000
**Willingness to pay to have a safer vehicle**	6,697,260	8,823,070	50,000	80,000,000	5,000,000	5,000,000

Table 2 containing results of maximum likelihood using 500 (5,000 IRR) for zero values and regression imputation for missing values, shows that WTP was significantly associated with gender, age, middle and high monthly income, daily payment for risk reduction, payment for time reduction, in buses, minibuses, and private cars drivers and among the occupants(table. 2).

**Table 2:** Results of maximum likelihood estimation using 500 (5,000IRR) for zero values and regression imputation for missing values.

Variable		Est.	S.E.	z-value	p-value
Intercept		9.6015	0.4674	20.539	P<0.001
**Gender(Male)**		**−0.5348**	**0.1353**	**−3.951**	**P<0.001**
**Age**		**0.0133**	**0.0043**	**3.043**	**P<0.001**
Education(High school & diploma)		0.1092	0.0866	1.259	NS
Family size	Less than 4	0.029	0.1006	0.288	NS
	equal to 4	−0.0149	0.0977	−0.153	NS
**Income**	**Middle income**	**0.5368**	**0.1905**	**2.817**	**P<0. 01**
	**High income**	**0.7031**	**0.2128**	**3.303**	**P<0.001**
Has had an accident		−0.06	0.0783	−0.767	NS
Log(kilometer moving)		−0.0812	0.0421	−1.924	NS
**Log(daily payment to injury reduction)**		**0.289**	**0.0423**	**6.823**	**P<0.001**
**Log(payment to time reduction)**		**0.0566**	**0.0143**	**3.947**	**P<0.001**
Health	Low	0.0359	0.1160	0.309	NS
	Middle	0.0486	0.0895	0.543	NS
**Road users**	**Bus**	**0.9438**	**0.1417**	**6.656**	**P<0.001**
	**Minibus**	**0.6822**	**0.1297**	**5.257**	**P<0.001**
	**Car**	**1.152**	**0.1653**	**6.966**	**P<0.001**
	**Occupants**	**0.4766**	**0.1561**	**3.053**	**P<0.001**
	Pedestrian	−0.0495	0.1496	−0.331	NS
Log(scale)		0.0493	0.0280	1.755	NS
N		782			
Log Likelihood		−10350.8			

Table 3, containing results of maximum likelihood using a value less than 1000 (10,000 IRR) for zero values and regression imputation for missing values, shows that WTP had a significant relationship with gender, age, middle and high monthly income, daily payment for risk reduction, payment for time reduction, in buses, minibuses, and private cars drivers and among the occupants (table. 3).

**Table 3:** Results of maximum likelihood using a value less than 1000(10,000 IRR) for zero values and regression imputation for missing values.

Variable		Est.	S.E.	z-value	p-value
Intercept		9.5931	0.4727	20.293	P<0.001
**Gender(Male)**		**−0.5353**	**0.1367**	**−3.915**	**P<0.001**
**Age**		**0.0133**	**0.0044**	**3.016**	**P<0.01**
Education(High school & diploma)		0.1084	0.0876	1.237	NS
Family size	Less than 4	0.0291	0.1018	0.286	NS
	equal to 4	−0.0151	0.0988	−0.153	NS
**Income**	**Middle income**	**0.5365**	**0.1927**	**2.785**	**P<0.01**
	**High income**	**0.7029**	**0.2152**	**3.266**	**P<0.01**
Has had an accident		−0.0618	0.0792	−0.78	NS
Log(kilometer moving)		−0.0806	0.0426	−1.89	NS
**Log(daily payment to injury reduction)**		**0.2895**	**0.0428**	**6.759**	**P<0.001**
**Log(pay to time reduction)**		**0.0568**	**0.0145**	**3.917**	**P<0.001**
Health	Low	0.0351	0.1173	0.3	NS
	Middle	0.0484	0.0905	0.535	NS
Road users	**Bus**	**0.9433**	**0.1433**	**6.583**	**P<0.001**
	**Mini bus**	**0.681**	**0.1311**	**5.193**	**P<0.001**
	**Car**	**1.1512**	**0.1671**	**6.888**	**P<0.001**
	**Occupants**	**0.4735**	**0.1577**	**3.003**	**P<0.01**
	Pedestrian	−0.051	0.1512	−0.337	NS
Log(scale)		0.0602	0.0286	2.103	NS
N		782			
Log Likelihood		−10080.7			

**Figure 4:**
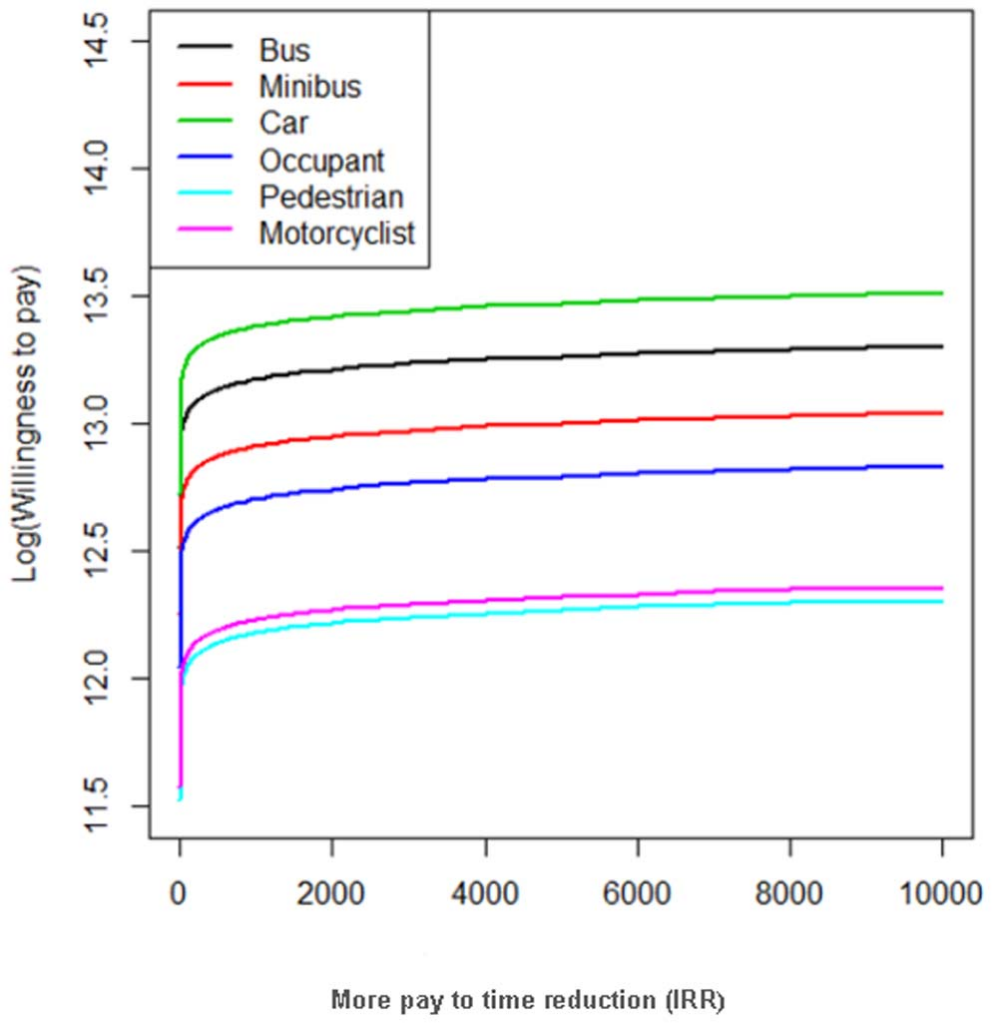
A predictive plot of logarithm of willingness to pay(IRR) against pay to time reduction for different road users (A man with high school or diploma education, family size less than 4, middle income, have an accident experience, trip mileage  = 30 km, more pay to time reduction = 2,000IRR, middle health.

Fig 4 shows a predictive plot (obtained by the results of the model using classical approach and zero values are assumed to be less than 1000(10,000 IRR) of the effect of pay to time reduction on the logarithm of WTP for different road users such as table 2. This plot is obtained for a man with high school or diploma education, family size <4, middle income, accident experience, daily trip distance  = 30 km, more pay to time reduction  = 2,000 IRR, and middle health.

This figure shows that the WTP pay for a car user with the above characteristics is more than that for other users, and is increased by more pay to time reduction (fig. 4).

## Discussion

Our estimate of the cost of each traffic fatality in Iran in 2013 was 19,713,584,906 IRR. Willingness to pay was higher among those who had extra payment for reducing trip time, and had more daily (charity) payment. Willingness to pay increased up to the age of 35, but gradually decreased from 35 to 55 and grew again after 55 years old. Willingness to pay was higher among the people who had middle and higher income than those with low income. It also had a significant relationship with age, gender, trip mileage, and all road users except pedestrians. More than four-fifths of the studied population traveled mostly for business. Willingness to pay to have a safer vehicle was higher than WTP for risk reduction.

In 2005, Bhattacharya used the WTP method to estimate the cost of one traffic injury in Delhi at US$150,000 [14] as in our research; Bhattacharya found that WTP increased with higher income and risk reduction. In Le H's study, the real value of accident cost was estimated using a method that reflected willingness of the society to pay. The statistical value of life avoided fatality in car was $1,874,000 and for motorcycle was $1,711,000 and value of avoided serious injury was $ 1,426,000 [15]. Pitel calculated the number and cost of injuries caused by traffic injuries using the WTP method in Canada and demonstrated that, between 1990 and 2010, accidents attributed to the consumption of alcohol and drugs caused 14,256 deaths, 841,004 injuries, and damage to 2,779,458 vehicles. Cost of the injuries was estimated at US$2,062,000,000 [16].

Our findings showed that WTP for reducing fatality risk was at its highest rate at the age of 35, gradually decreased up to the age of 55, and then increased again after 55 years old. Similarly, Van showed that the statistical value of life was higher at age 40 than ages 20 or 65 years old. Van speculated that the explanation for this effect was that income levels increase between 20 and 40 years old, but people are less afraid of dying at the age of 40 than they are at 20. Since children typically possess little money, their WTP is at the lowest rate [17]. The difference between the age of WTP in our study and others might be because of the greater job stability and higher income of people at age 35 than other ages; so, they declare a real amount of WTP. Also, better perception of risks due to life experience (and possibly direct experience of injuries and injury costs) are the reasons why people aged 35 reports a higher WTP. As other authors have found, at less than 35 years old, lower understanding of risk and less payment ability translate into low WTP.

Calculating the statistical value of life is one of the most important components in determining the value of risk reduction [18]. Calculating the statistical value of life is a key component in general policymaking, used frequently in the evaluation of efficiency in environmental projects and field-making which are effective in death. In our own study, the cost of traffic injuries and deaths in Iran totaled US$390,048,341,074 for 2013, over 6·5 times the sum estimated using a human capital approach in 2012.^4^ As noted in the introduction, this method relies on data which is usually under-reported, and neglects several important sources of information. Children and the elderly (who generate very little capital production) are not included in this method, while the social and medical costs of accidents are ignored [19].

During the past recent decades many countries have used the WTP approach to estimate the costs of traffic accidents, Willingness to pay is the value considered by people for social death reduction. Several studies have emphasized the accuracy of this method [20], [21]. The prevention value of traffic injuries is the amount which society prepared to pay to prevent injuries; although it seems theoretical, it is a better reflection of the true economic cost of death and injury. People may not be able to say how much their lives are worth, but they can estimate how much they would pay to reduce risk. If WTP is considered to aim at reduced risk, a sign for the statistical value of life would be obtained [22]. Value of risk change or WTP is based on fundamental assumptions, and shows that the adopted decisions on resource allocation in the government sector should reflect citizens' demands and preferences. The value allocated for improving road safety (risk reduction) is the total amount paid by people for preparation [23]–[24].

Abelson presented a monetary value for life survival by expressing some examples and contrasts. One-sixth of gross national income in Sydney of Australia is spent on health and injury prevention [25]. Ideally, all countries should conduct a WTP study to calculate the value of statistical life in traffic injuries before making any kind of investment in road safety [26]. Hensher showed the statistical value of life was an efficient method for the economic analysis of safety benefit and promotion of road environment [27]. Other studies have demonstrated that preference expression studies are valuable in policymaking [11], [28]–[30]. New approaches are being implemented for calculating the statistical value of life. However, no improvement has been made in calculation methods for lost output, medical costs, and other costs (human capital model) [22], [31].

In Le H's study, the cost of traffic accidents was estimated using a method that reflected willingness of the society to pay. Two methods of contingent valuation and stated preference for WTP were used for deducing accident costs [13]. The current study used the revealed preference method in addition to the methods employed in Le H's study. Our population was more willing to pay in order to increase safety than reduce risk. It is worth noting that the studied population was more willing to pay in the cases, in which family safety was guaranteed by adding safety devices. In theoretical terms, WTP is an accurate method for determining the cost of injuries; it is conceptually correct and provides a better reflection of the social value of safety than other methods [32].

The innovative aspect of our study was its use of the three methods of contingent valuation, stated preference, and revealed preference. No previous research has involved the simultaneously use of these three methods. In addition, we used a large sample size; also, Weibull models were used for modeling WTP data. We used three different methods to impute missing values (mean, median, and regression methods imputation; similar studies did not mention missing values or their means of dealing with them. All the variables influencing WTP were extracted from previous published research and analyzed in the current study. Considering all the effective variables in willingness to pay, the model proposed in our study can be used at local, regional, and national levels. One of the key points was risk perception; so, subjects should have at least high school education and be in the age group of 18–65 years old. These could be considered the study's limitations.

## Conclusions

Willingness to pay was significantly associated with age, gender, income, daily payment for reducing injury risk, payment for reducing trip time, and type of road user (occupants or public vehicle drivers). The costs of traffic injuries accounted for 6·64% of Iran's gross national income in 2013, a rate much higher than the global average. Policymaking and resource allocation based on scientific evidence about the cost of traffic injuries in Iran could result in significant economic savings. Our results, and those of similar studies in other countries, suggest that willingness to pay research is an important prerequisite to investment in road safety measures.
